# The Effects of Different Hygiene Instrumentations on Titanium Implant Fixture Surfaces

**DOI:** 10.7759/cureus.74645

**Published:** 2024-11-28

**Authors:** Hareem Ghaffar, Haslina Taib, Mohamad Arif Awang Nawi, Akram Hassan

**Affiliations:** 1 School of Dental Sciences, Universiti Sains Malaysia, Kota Bharu, MYS

**Keywords:** airflow, hygiene instruments, laser, scanning electron microscope (sem), titanium brush, titanium implant fixtures

## Abstract

Background and objective

Applying different hygiene tools for implant maintenance alters surface configurations, impacting bacterial adhesion on titanium implant surfaces and potentially leading to peri-implant diseases. This study aimed to assess the alterations in surface topography of titanium implant fixtures after utilizing hygiene instruments such as airflow; erbium, chromium-doped: yttrium, scandium, gallium, and garnet (Er, Cr: YSGG) laser; and titanium brush, under scanning electron microscope (SEM) observation.

Materials and methods

We employed an experimental laboratory study design for this research, involving 20 MegaGen ST titanium implant fixtures (MegaGen Implant Co., Ltd., Seoul, Republic of Korea). These were classified into four groups: untreated/control fixtures (n=5); fixtures treated with airflow using sodium bicarbonate powder (n=5); fixtures treated with an Er, Cr: YSGG laser system at 1.5 W power and a laser pulse of 30 Hz (n=5); and fixtures treated with titanium brush operating between 300 to 1000 rpm (n=5). All fixtures were then rinsed with normal saline, fixed with 2.5% glutaraldehyde, and observed under SEM.

Results

In the SEM analysis, at 50x magnification, there were no differences between the fixtures. However, at 1000x, 2000x, and especially at 5000x magnification, the control group surfaces appeared to have no obvious change and were quite similar, with clearer surface details. The airflow group exhibited smoother surfaces with less complex and more organized structures. The laser group displayed a more irregular and haphazard structure, revealing a rougher topography. The titanium brush group showed the areas of the implant fixture surfaces appearing smoother and flatter.

Conclusions

In this SEM study, the titanium brush group yielded the smoothest surfaces and the most favorable overall outcomes, highlighting its efficacy.

## Introduction

Dentistry has witnessed incredible advancements recently, and numerous innovative methods have emerged to enhance the quality of life of patients who undergo dental procedures. Adults often experience tooth loss, whether partially or completely, resulting from various reasons such as trauma, congenital absence, decay, or gum disease. This loss significantly impacts both the function and appearance of one's smile. Fortunately, several solutions exist for individuals with missing teeth, with dental implants being the preferred treatment for tooth replacement.

The main concern regarding plaque removal from implant surfaces is the risk of potential damage to the implant surface. If plaque accumulates and persists, inflammation around the implant can swiftly spread to the surrounding bone [1]. This escalation of inflammation could lead to bone resorption, hindering the process of osseointegration. The biological side effects of dental implants that result from infections triggered by bacterial biofilm and subsequent inflammation of the soft tissues and bone surrounding implants are known as peri-implant diseases, including peri-implant mucositis and peri-implantitis [2,3]. Dental biofilm, commonly called plaque, is a dense, sticky layer of bacteria and their byproducts that adheres to the surfaces of teeth, dental implants, gums, and other areas in the mouth. It forms when microorganisms in the mouth accumulate on the tooth or dental implant surfaces and form a protective layer primarily comprising bacteria, saliva proteins, and food particles. It is highly structured, with bacteria embedded in a matrix of extracellular polymeric substances (EPS), allowing it to adhere firmly to tooth or dental implant surfaces, especially along the gum line. Plaque develops from these oral microbiota accumulations [4]. Peri-implant mucositis around dental implants can be largely reduced by reducing plaque build-up [5].

Regular maintenance of dental implants by both patients and dental healthcare professionals is essential for preventing peri-implant diseases [6]. The primary goal is to minimize plaque buildup on dental implant surfaces. Patient home care involves activities like tooth brushing, using interdental cleaning tools such as floss and interdental cleaners, and employing locally applied treatments like chlorhexidine gluconate and water flossers such as Hydro Floss® [7]. Professional implant hygiene care includes various methods such as laser, plastic curette, titanium curette, air-powder abrasive system, and rubber cup with non-abrasive paste [7,8]. However, these hygiene care instruments can potentially modify the implant surface. As a result, the surface profile and roughness produced by these modalities may significantly affect the newly formed biofilm, thereby playing an important role in peri-implant health maintenance [9].

Dental implants' sustained effectiveness largely depends on regular maintenance and follow-up procedures. General dentists are becoming more and more responsible for providing this continuing care as dental implants become more widespread. The structure of the implant surface and the cleaning techniques used significantly influence how well the implant surface is cleaned [10]. Plaque and calculus must be effectively removed from the implant surface by the maintenance instruments to minimize any possible damage. Implant surfaces are significantly altered by traditional sonic and ultrasonic scalers [11]. Therefore, recommendations have been made to use rubber polishing, graphite or nylon instruments, and plastic curettes. However, as per the literature, there is currently no widely accepted method for cleaning and polishing oral implants without potentially causing harm to the implant surfaces.

This study investigated the impact of three different types of tools used for implant maintenance. It aims to provide a new understanding and perspective on how these tools affect the roughness of titanium surfaces. We believe this knowledge will aid dentists in choosing the most effective and least intrusive methods to maintain the health of implants. Therefore, the study's objective was to utilize a scanning electron microscope (SEM) to analyze the surface topographies of titanium implant fixtures in groups that were untreated and those treated with an airflow; erbium, chromium-doped: yttrium, scandium, gallium, and garnet (Er, Cr: YSGG) laser; and titanium brush.

## Materials and methods

This research employed an experimental laboratory study design. The PS Power and Sample Size Calculation software [12] was used to determine the sample size based on the comparison of two means. A type one error of 0.05 and a study power of 0.8 were set. The expected change in mean surface roughness, based on previous study results, was 0.20, with a standard deviation (SD) of 0.06 [8]. The calculated sample size was 73 fixtures per group. However, as this was an exploratory study and due to limitations in cost and time, the sample size was reduced to five fixtures per group.

Twenty MegaGen ST titanium implant fixtures (MegaGen Implant Co., Ltd., Seoul, Republic of Korea) were used for this study. Five titanium implant fixtures were randomly classified into four different groups: untreated/control group (n=5); airflow group (n=5); Er, Cr: YSGG laser system group (n=5); and titanium brush group (n=5). They were mounted on a 2 x 2 x 2 cm stone stand for the hygiene instrumentations.

Hygiene instrumentations

(i) Control Group

The samples were taken directly from sealed sterile packages and no further treatment was applied to these samples.

(ii) Airflow Group

Samples were treated with an airflow (Mectron Starjet) using NaCOH_3_-based powder (Mectron prophylaxis powder soft). The device was connected to the dental unit turbine. Fixtures were treated using high-pressure air and water jets in combination with a specially formulated powder (sodium bicarbonate). An airflow was performed for 20 seconds at a distance of 5 mm from the surfaces by moving the tip of the handpiece at an inclination of 30 degrees with pendular brushing movements [13]. Standard water pressure (from one to three bars) and standard air pressure (two to three bars) were used for the dental unit turbine, as recommended by the manufacturer.

(iii) Er, Cr: YSGG Laser Group

The laser system used was Er, Cr: YSGG (Waterlase ExpressTM, Biolase Technology, San Clemente, CA). A wavelength of 2780 nm was utilized with a radical firing perio tip (RFTP5-14mm, Biolase). The laser settings were configured to a power output of 1.5 W. During application, the laser tip was positioned parallel to the implant surface at a focal distance of 0.5 mm for 120 seconds. The laser pulse frequency was set at 30 Hz, accompanied by air and water irrigation at 50% of the maximum flow rate [14].

(iv) Titanium Brush Group

A titanium rotary brush (Ti brush, Titanium Brush DTST-6352, 38 mm; MegaGen Implant Co., Ltd.) was used with a force of 0.25 N. It began at 300 rpm and could increase to 1000 rpm as needed. It was compatible with a slow-speed implant handpiece. The instrumentation was performed with water irrigation in a rotative direction on the surfaces [15].

All fixtures from each group were thoroughly rinsed with normal saline, fixed in 2.5% glutaraldehyde, and then examined using SEM.

Scanning electron microscope (SEM)

**Figure 1:**
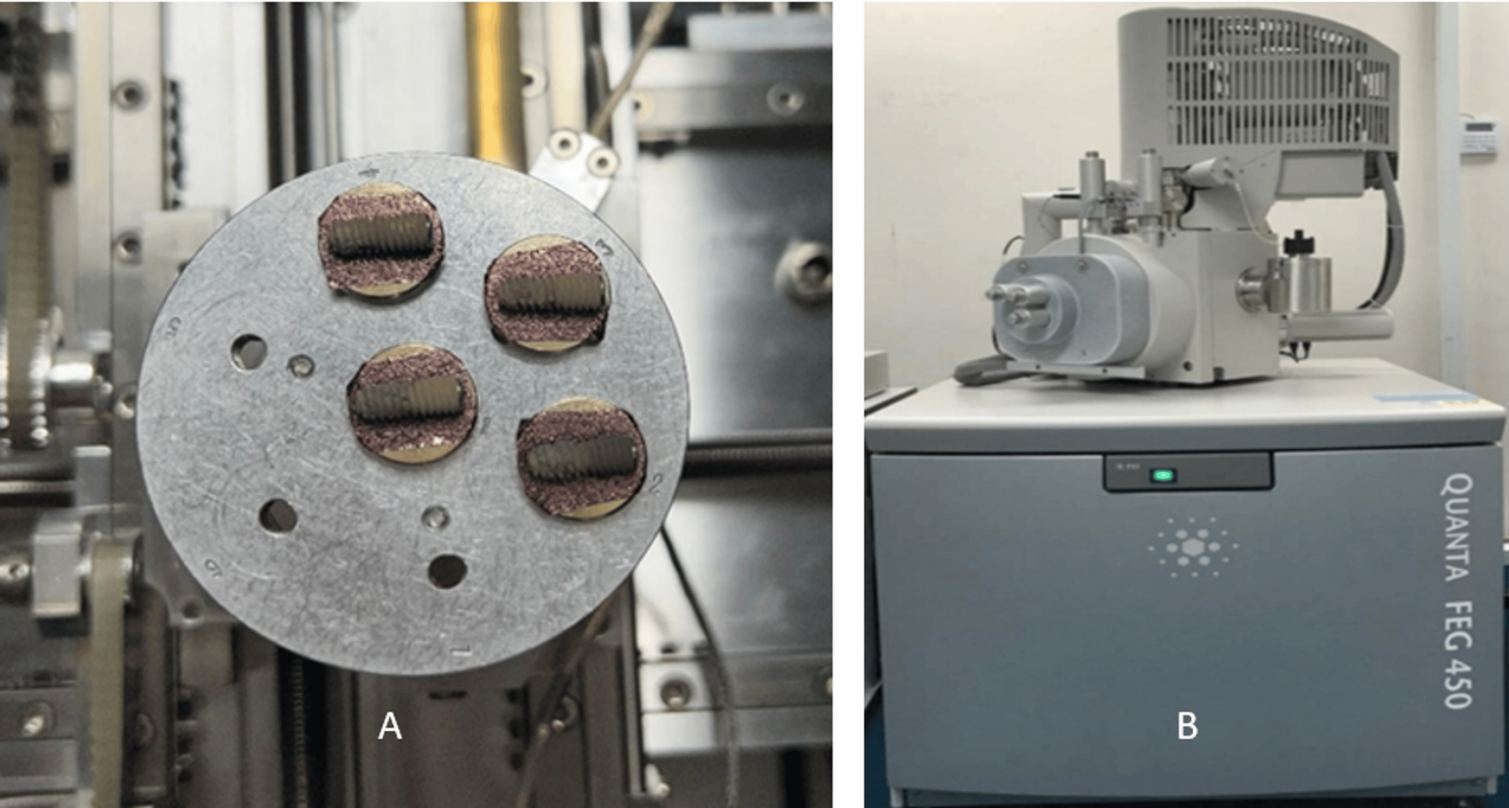
(A) SC005 Leica Sputter Machine was used to coat each sample with gold powder prior scanning. (B) Surface analysis of the fixture was done using Quanta FEG 450

A random sample from each group was selected and analyzed with an SEM to evaluate surface roughness topography. The samples shown in Figure 1A were coated with gold powder using a SC005 Leica Sputter Machine to improve visibility during SEM analysis. The samples were subsequently mounted onto a stub and analyzed as shown in Figure 1B, using a FEG Quanta 450 for high-resolution imaging, with careful selection and standardization of the scanning surface. It was occupied with a high-resolution field emission column, optimized for high brightness/high, low, and extended low vacuum, thus producing clearer, less electrostatic distorted images with spatial resolution down to 1.4 nm, three to six times better than conventional SEM.

The magnifications selected were 50x, 1000x, 2000x, and 5000x thoroughly over the selected area, and were compared with the untreated (control) group. The captured image was viewed and edited using software (XT microscope control) in a computer system attached to the SEM machine.

## Results

**Figure 2:**
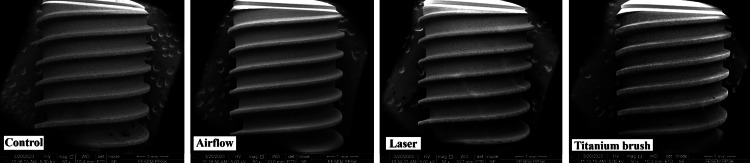
Surface topography at 50x magnification in the four groups Er, Cr: YSGG: erbium, chromium-doped: yttrium, scandium, gallium, and garnet

At 50x magnification (Figure 2), the surfaces of implant fixtures appeared quite similar in all four groups. There were no obvious surface changes between the control; airflow; Er, Cr: YSGG laser; and titanium brush groups.

**Figure 3:**
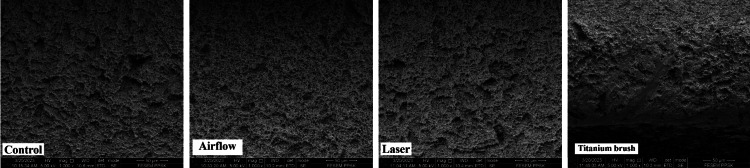
Surface topography at 1000x magnification in the four groups Er, Cr: YSGG: erbium, chromium-doped: yttrium, scandium, gallium, and garnet

**Figure 4:**
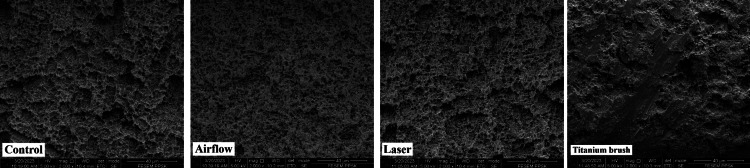
Surface topography at 2000x magnification in the four groups Er, Cr: YSGG: erbium, chromium-doped: yttrium, scandium, gallium, and garnet

**Figure 5:**
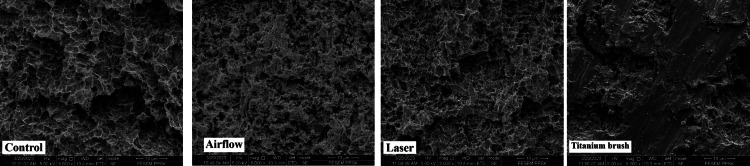
Surface topography at 5000x magnification in the four groups Er, Cr: YSGG: erbium, chromium-doped: yttrium, scandium, gallium, and garnet

The surface topography was subsequently analyzed at 1000x, 2000x, and 5000x magnifications. In the control group, at magnifications of 1000x, 2000x, and 5000x (Figures 3-5), there were no noticeable alterations in the surface topography of the fixtures, suggesting that they likely appeared similar under clearer surface magnifications. The airflow group at magnifications of 1000x, 2000x, and 5000x (Figures 3-5) showed smoother surfaces with less complex and organized surface structures due to the polishing effect of the powder which smoother the roughed surfaces' pits compared to the control group. The laser group at magnifications of 1000x, 2000x, and 5000x (Figures 3-5) showed a more complex, haphazard structure as the surfaces showed a more roughed topography, especially at 5000x. The titanium brush showed the most effective results of all magnifications as it showed the areas of the implant fixture surfaces that were smoother, and flatter.

## Discussion

It is well known that even though implants are prosthetics, they perform the same functions as natural teeth in the oral cavity and are susceptible to bacterial and plaque buildup. Literature has made it abundantly evident that there is currently no cleaning and polishing procedure for oral implants that can be used anywhere without harming the surfaces of the implants [16,17]. Many techniques such as air abrasives, plastic instruments, and polishing rubber cups have been proposed, but there is no clear-cut best practice as yet [18,19]. Using hygiene instruments during supportive implant therapy affects the surface modification of implant surfaces as it affects the rates of bacterial adhesion. These therapies may harm the titanium oxide stable protective coating on implant surfaces, despite being the recommended approach for addressing peri-implant diseases [20]. To achieve an anti-adherent and plaque-reducing effect, every micro-structured component that encounters the oral cavity needs to be highly polished.

This study engaged in a comparison between three hygiene instrumentation methods on titanium implant fixtures under SEM. For this study, the magnification of 50x, 1000x, 2000x, and 5000x were used. The findings indicated that at a magnification of 50x, all groups exhibited the same surface topography, as compared to the control/untreated group, which displayed typical sharp-edged elevations. However, at magnifications of 1000x, 2000x, and 5000x, the polishing effect of the powder led the airflow group to exhibit smoother surfaces with simpler and more organized structures than the control group, effectively reducing the pits found on rougher surfaces.

Sawai et al. [21] found that with the varied rpm, pressure or load, time spent polishing each surface, and coarseness of the polishing pastes used in different studies, it was difficult to directly compare the results of previous studies on the impact of airflow with powder groups to the alterations of implant surfaces. In contrast with Ichioka et al. [22], they revealed that there were no noticeable changes to the titanium surface when employing airflow techniques. In the study by Tavakoli et al. [23], titanium brushes and curettes produced a larger change in roughness in comparison to the control group, even though they produced a smoother surface. After polishing with the airflow, the surface was the roughest, but the group that received airflow also showed the least changes (in comparison to the control group).

Inconsistent with the previous studies, studies utilizing SEM have documented the effectiveness of Er, Cr: YSGG laser irradiation as an implant decontamination method without producing any surface modification [14,24,25]. The distance of Er Cr: YSGG laser irradiation in this study was similar, but the laser tip, duration, power, and scanning technique utilized in the previous research varied [24]. It was demonstrated that the 1.5 W power setting did not create significant changes in surface micro-roughness, similar to the power level and tip type [14], where they used radial firing tips to treat failed implants. Nevertheless, the SEM result for the Er, Cr: YSGG laser group was noted to produce surface roughness, as the surfaces exhibit a more rugged topography, particularly at 5000x. This laser group displayed a more intricate, disorganized structure. This observation is consistent with Chegeni et al. [26], who found that implant surface modifications showed greater damage, such as melting effects, when the energy per pulse was increased to 84 mJ (2.5 W/30 Hz), compared to an Er, Cr: YSGG laser with side-firing and conical tips operating at 50 mJ (1.5 W/30 Hz).

Titanium surfaces are altered when instrumented either with a nylon brush or a metal brush. Hence, nylon or metal brushes should be used with caution to avoid damaging the implant fixture/abutment surface [27]. The titanium brush reveals the areas of the implant fixture surfaces like leveling of typical sharp edges and flatter, smoother surfaces, yielding optimum results across all magnifications [15]. Al-Hashedi et al. [28] evaluated the ability of clinically available methods, such as metal and plastic curettes, titanium brushes, and the erbium-doped yttrium aluminum garnet (Er: YAG) laser to decontaminate implant surfaces. The surface morphology, chemical composition, and properties of the machined titanium discs were analyzed before and after the contamination of oral biofilms by SEM and X-ray photoelectron spectroscopy. Titanium brushes were found to be more effective than curettes (metal or plastic) and the Er: YAG laser in decontaminating titanium implant surfaces, although neither technique was able to fully remove surface contamination. These results align with the study by Toma et al. [29], which showed better results in the decontamination of the implant surface in the group using titanium brushes vs. the plastic curettes group or the abrasive air group.

In line with previous research, our findings indicate that the titanium brush produces smoother and flatter surfaces, effectively decontaminating titanium implant surfaces.

Limitations

This study has several limitations. Firstly, it involved a one-time instrumentation assessment, and the results may vary in future applications within real clinical settings. Additionally, previous studies have indicated that titanium disks were typically utilized as samples due to their more homogeneous and flat surfaces, which aid in reducing errors during instrumentation manipulation. Finally, an issue arose regarding the number of uses for the titanium brush. Previous research employed a single-use titanium brush for implant decontamination. However, according to the manufacturer’s guidelines, the MegaGen® titanium brush can be reused multiple times, as long as there are no visible signs of corrosion, cracks, or pinholes. The impact of multiple uses in the context of routine professional mechanical plaque removal remains unclear and speculative.

## Conclusions

In this study, the surface topography treated with the laser group showed the least effective results with a more complex and disorganized structure compared to the other groups. The airflow group seemed to have generalized micropores due to the high velocity of airflow and powder, and the titanium brush group showed the most effective results as the surfaces were flatter and smoother and showed fewer complex structures which will be less prone to implant health and periodontal diseases.
